# Supplementary material for: SYK-623, a δ Opioid Receptor Inverse Agonist, Mitigates Chronic Stress-Induced Behavioral Abnormalities and Disrupted Neurogenesis
Source: J Clin Med. 2024 Jan 21;13(2):608. doi: 10.3390/jcm13020608 (PMC10817044; doi:10.3390/jcm13020608)
Supplement: Supplementary file 1 [file jcm-13-00608-s001.zip › jcm-2820330-supplementary.pdf]

Supplemental Table S1. Treatment groupings

Cohort 1	Number of mice	
	Total	Fixed for immunohistochemistry
Treatment		
Non-stress + vehicle (saline)	10	5
ACMS + vehicle	10	5
ACMS + imipramine	10	5
Non-stress + vehicle (10% DMSO-containing saline)	10	10
ACMS + vehicle (10% DMSO-containing saline)	10	10
ACMS + SYK-623	10	10
Non-stress + vehicle (saline)	10	10
ACMS + vehicle	10	10
ACMS + NTI	10	10

Cohort 2	Number of mice	
	Total	Tissue sampling for immunohistochemistry
Non-stress + vehicle (10% DMSO-containing saline)	9	9
ACMS + vehicle (10% DMSO-containing saline)	9	9
ACMS + SYK-623	9	9

Animals with following conditions were excluded from each experiment.
All experiments: Body weight loss exceeding 15% compared to the start of the experiments.
Tissue weight and immunohistochemical staining: Partial loss or damage of tissue during extraction. Residual blood in the brain post-fixation (indicating insufficient fixation).
Tail suspension test: Escape by climbing their tail or adhesion tape. Fall due to the peeling of adhesive tape.
Y-maze: Total arm entries less than 9 times. Exploration in the same direction.
Novel arm recognition test (Modified Y-maze): Total arm entries less than 9 times.
Novel object recognition test: Less than 20 seconds of exploration time towards the objects during the training session, or a bias in interest towards the object on one side.
Open field test: Total distance of less than 1000 cm.
